# Executive attention networks show altered relationship with default mode network in PD

**DOI:** 10.1016/j.nicl.2016.11.004

**Published:** 2016-11-05

**Authors:** Peter Boord, Tara M Madhyastha, Mary K Askren, Thomas J Grabowski

**Affiliations:** a Department of Radiology, University of Washington, United States; b Department of Neurology, University of Washington, United States

**Keywords:** ANT, attention network test, DAN, dorsal attention network, DMN, default mode network, FC, functional connectivity, FPN, frontoparietal task control network, HC, healthy controls, lIPS, left intraparietal sulcus, PD, Parkinson's disease, rFEF, right frontal eye field, rIPS, right intraparietal sulcus, RSN, resting state networks, rSPL, right superior parietal lobule, TPN, task positive network, VAN, ventral attention network, Executive attention, Default mode network, Parkinson's disease, Attention network test, Functional connectivity

## Abstract

Attention dysfunction is a common but often undiagnosed cognitive impairment in Parkinson's disease that significantly reduces quality of life. We sought to increase understanding of the mechanisms underlying attention dysfunction using functional neuroimaging. Functional MRI was acquired at two repeated sessions in the resting state and during the Attention Network Test, for 25 non-demented subjects with Parkinson's disease and 21 healthy controls. Behavioral and MRI contrasts were calculated for alerting, orienting, and executive control components of attention. Brain regions showing group differences in attention processing were used as seeds in a functional connectivity analysis of a separate resting state run. Parkinson's disease subjects showed more activation during increased executive challenge in four regions of the dorsal attention and frontoparietal networks, namely right frontal eye field, left and right intraparietal sulcus, and precuneus. In three regions we saw reduced resting state connectivity to the default mode network. Further, whereas higher task activation in the right intraparietal sulcus correlated with reduced resting state connectivity between right intraparietal sulcus and the precuneus in healthy controls, this relationship was absent in Parkinson's disease subjects. Our results suggest that a weakened interaction between the default mode and task positive networks might alter the way in which the executive response is processed in PD.

## Highlights

• PD shows elevated task positive network (TPN) activation in an executive response.
• These regions show lower functional connectivity (FC) with the default network (DN).
• TPN activation and TPN-DN FC are associated in controls, but dissociated in PD.

## 1 Introduction

Attention dysfunction is commonly present in people with Parkinson's disease (PD) without dementia (Aarsland et al., 2010), and bears a significant impact on quality of life, with subjects finding difficulty maintaining concentration (Barone et al., 2009), and increasing evidence suggesting a role of attention dysfunction in falls and gait disturbance (Amboni et al., 2013). The precise nature and neural correlates of attention dysfunction in PD are yet to be fully elucidated. Early work suggested behavioral deficits only manifest when task demands exceed attention resources in the frontal cortex under novel, non-routine conditions (Stam et al., 1993), or for tasks that depend on internal cues (Brown and Marsden, 1988). It was hypothesized this might arise from degeneration of dopaminergic mesocortical innervation of the frontal cortex (Stam et al., 1993). Further work supported a genetic influence affecting dopaminergic frontostriatal networks as well as a frontoparietal network (Williams-Gray et al., 2008). PET imaging studies in PD demonstrate glucose hypometabolism in frontal and parietal areas and imply an important component of cortical dysfunction (Klein et al., 2010, Meles et al., 2015), which is further supported by findings of cortical thinning in PD (Madhyastha et al., 2015b). More recent work suggests that degeneration of the ascending cholinergic system might also impair attention networks in PD (Sarter et al., 2014).

Functional neuroimaging has long used task-based fMRI to study attention (Posner, 2012). Fan and colleagues (Fan et al., 2005) designed an Attention Network Test (ANT) to measure three aspects of attention: alerting (achieving and maintaining an alert state), orienting (selecting the spatial location of sensory input), and executive control (resolving conflict; (Posner, 2012)). Brain networks subserving these attention systems have been postulated to relate to different neuromodulators, with the alerting network modulated by norepinephrine, orienting by acetylcholine, and the executive network by dopamine (Posner, 2012). Another model derived from task-based fMRI data divides attention into partially segregated dorsal and ventral frontoparietal networks, serving top-down and bottom-up information processes respectively (Corbetta and Shulman, 2002). These networks have more recently been observed in the inter-regional correlation structure, or functional connectivity, of spontaneous resting state brain activity (Fox et al., 2006). Within this system level organization of brain function, activation of the dorsal attention network (DAN) correlates with deactivation of a default mode network (DMN) (Fox et al., 2005), while switching between the DAN and DMN is associated with a frontoparietal task control network (FPN) (Spreng et al., 2010, Vincent et al., 2008). It is not known whether alerting/orienting/executive “networks” have such spatially resolved components.

A number of recent studies have observed resting state functional connectivity (rsFC) changes in brain networks of PD subjects (Prodoehl et al., 2014, Szewczyk-Krolikowski et al., 2014, Wu et al., 2015). However, few studies have examined task activated attention networks and their possible relationship with rsFC changes in resting state networks in the same subjects. We sought to address this gap in knowledge by analyzing brain activity at rest and during the ANT task examining alerting, orienting, and executive components of attention.

## 2 Methods

### 2.1 Participants and dopaminergic medication

Twenty five subjects with non-demented PD and twenty one healthy controls (HC) participated in this study (Madhyastha et al., 2015a). Subject characteristics are summarized in Table 1. PD subjects were recruited from a larger parent study where they underwent extensive clinical examination and neuropsychological assessment (Cholerton et al., 2013). In brief, information regarding activities of daily living were garnered from a clinical interview of the patient and caregiver. Subjects were diagnosed in a diagnostic consensus conference conducted in the clinical core of the Pacific Northwest Udall Center (NS P50 062684). Subjects were diagnosed with either no cognitive impairment (PD-NCI), mild cognitive impairment (PD-MCI), or dementia (PDD). PDD subjects met published diagnostic criteria (Emre et al. 2007). The present study included 14 PD-MCI and 11 PD-NCI subjects from the parent study. The PD-MCI subjects did not differ from PD-NCI on cognitive test scores, but were judged to have declined from premorbid abilities based on education and the Shipley-2 vocabulary score. The reader is referred to the parent study report for full characterization of the sample (Cholerton et al., 2013).

Groups differed significantly on the UPDRS Part III (motor subscale), t(44) = − 11.8, *p* < 0.001, and in proportion of male, t(44) = − 2.05, *p* = 0.047, two-tailed *t*-test, but did not differ significantly in education, age, handedness, or MoCA (Montreal Cognitive Assessment score). The exclusion criteria of the study were no history of primary neurodegenerative disease other than idiopathic PD, moderate to severe dyskinesia, a MoCA score < 23, head trauma, stroke, severe cardiovascular disease, brain surgery, and contraindications for MRI. An additional four subjects were excluded from the study for falling asleep during functional scans, becoming claustrophobic in the scanner, being unable to perform the task, or voluntarily withdrawing from the study. Twenty three subjects were receiving daily administration of dopaminergic antiparkinsonian (DA) medication, and had received medication during their prior neuropsychological assessment (Cholerton et al., 2013). To compare our functional outcomes with results from the parent study, subjects were maintained on their daily DA regimen. The study comprised of two sessions, with subjects given their dosage one hour prior to the start of fMRI data acquisition for both sessions. The sessions were conducted at the same time of day and were separated by one to three weeks. The study was approved by the University of Washington Institutional Review Board. All participants provided written informed consent.

### 2.2 Study design

**Fig. 1:**
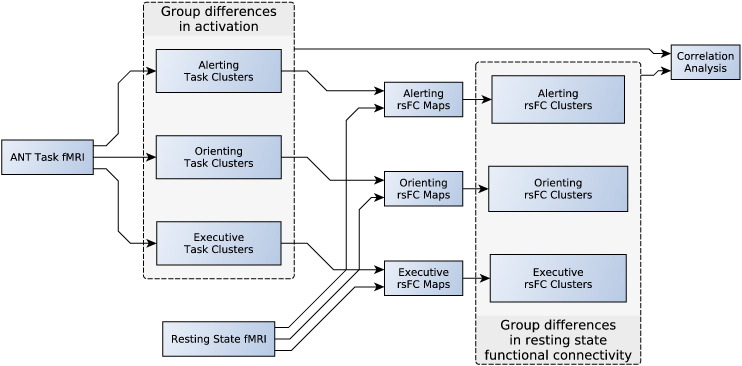
Study Design. The ANT fMRI task creates maps of group difference task clusters for each attention network. The resting state time series in each task cluster generates rsFC maps, which are compared across groups, generating rsFC clusters. ANT, Attention Network Test; rsFC, resting state functional connectivity.

The main purpose of the study was to identify group differences in attention networks, using both task activated and resting state fMRI. The design of the study is illustrated in Fig. 1. Imaging data was collected over two sessions, with each session comprised a resting state fMRI scan, followed by an fMRI task (Attention Network Test; ANT), and structural scans. The fMRI task data was analyzed first to identify brain regions (task clusters) where the groups significantly differed in activation for each attention component (alerting, orienting, executive). We then asked the question if there were any group differences in rsFC with those regions that differed in activation during the attention task. This was addressed by averaging the resting state fMRI time series in each task cluster; correlating this with the resting state fMRI time series at each voxel throughout the brain; and comparing the rsFC maps across groups. rsFC maps were generated and compared for each cluster for each attention component. This yielded brain regions (rsFC clusters) where the groups significantly differed in rsFC with the task clusters. Correlation analysis was then performed to assess for any group change in linear relationships between activation changes and rsFC changes.

### 2.3 Attention network test

**Fig. 2:**
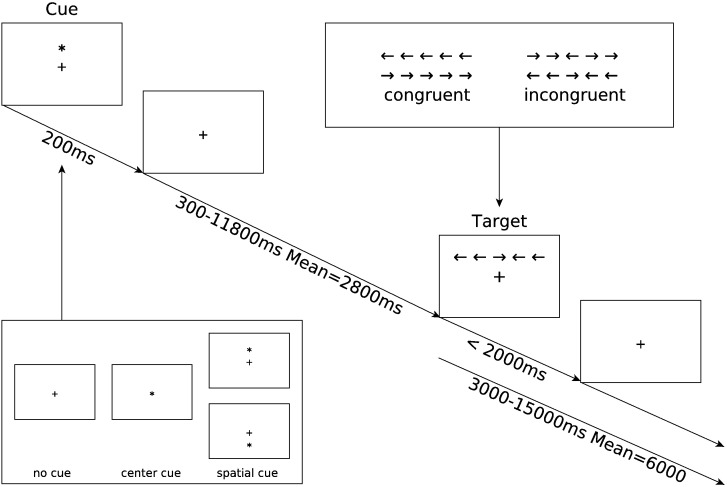
Attention network test schematic showing the timing and different conditions for each trial. A trial begins by presenting one of three cue conditions for 200 ms (no cue, center cue, spatial cue). A variable delay period of 300 to 11,800 ms elapses before one of two target conditions is presented (congruent, incongruent). The target disappears after 2000 ms, or when a response button is pressed. From the moment the target appears the trial continues for a variable delay period of 3000 to 15,000 ms.

All subjects performed the ANT (Fan et al., 2005) while in the MRI scanner. The ANT combines cues and targets within a single reaction time task (Fig. 2) to measure the efficiency of the alerting, orienting, and executive attention networks. We replicated the fMRI implementation of the ANT as described by Fan et al. (2005), and described here in brief. Subjects performed two scanning sessions one to three weeks apart. The purpose of acquiring two sessions was to serve as a baseline measurement for a third session taken after a cholinergic intervention, results of which are to be reported separately. Each session comprised of six separate runs, where subjects performed two buffer trials (discarded prior to analysis) followed by 36 reaction time trials (a total of 432 trials per subject). Trials consisted of a visual cue stimulus, a variable delay period, and a target stimulus. The intertrial period was also jittered. A fixation cross was presented in the center of the screen for the entire length of the run, with cue and targets superimposed. The target presented a row of five horizontal black arrows with either all arrows in the same direction (left or right; congruent condition) or with the center arrow in the opposite direction to the flanking arrows (incongruent condition). Subjects were required to press a button in their left or right hand according to the direction of the center arrow. The row of target arrows were positioned pseudorandomly with half above and half below a center fixation cross. The cue stimulus consisted of either no cue (no cue condition), a center asterisk (center cue), or an asterisk placed above or below the center fixation cross (spatial cue), in the same location as the subsequent target stimulus. Behavioral and imaging contrasts were then constructed to probe the efficiencies of the three attention networks:

Alerting effect = No cue response–Center cue response

Orienting effect = Center cue response–Spatial cue response

Executive effect = Incongruent target response–Congruent target response

Attention contrasts were calculated for reaction time, accuracy and fMRI activation.

### 2.4 Analysis of variance (ANOVA)

We performed a repeated measures ANOVA, using the GLM (general linear model) procedure in SAS Studio 3.4 (Statistical Analysis System, SAS Institute). For each subject, reaction time and accuracy were averaged using data from both sessions one and two. Separate ANOVA were used for reaction time and accuracy measures. Each ANOVA had a between-subject factor of group (PD, HC) and within-subject repeated measures factors of cue (No cue, Center cue, Spatial cue), and target (Congruent, Incongruent). The effects of age and years of education were removed by including these values as covariates of no interest in the GLM. Separate contrasts were specified for the alerting, orienting, and executive effects. Post hoc Scheffé tests were used to test for differences between groups for each test condition while controlling for multiple comparisons. Effects were considered significant when tests showed a *p*-value < 0.05.

### 2.5 MRI acquisition

Data were acquired using a Philips 3T Achieva MR System (Philips Medical Systems, Best, Netherlands, software version R2.6.3) with a 32-channel SENSE head coil. Each session included structural and functional scans. Whole-brain axial echo-planar images were collected parallel to the AC-PC line for a single resting state run and six task runs (43 sequential ascending slices, 3 mm isotropic voxels, field of view = 240 × 240 × 129, repetition time = 2400 ms, echo time = 25 ms, flip angle = 79°, SENSE acceleration factor = 2). Run duration was 300 volumes (12 min) for the resting state run and 149 volumes (5.96 min) for each task run. A sagittal T1-weighted 3D MPRAGE (176 slices, matrix size = 256 × 256, inversion time = 1100 ms, turbo-field echo factor = 225, repetition time = 7.46 ms, echo time = 3.49 ms, flip angle = 7°, shot interval = 2530 ms) with 1 mm isotropic voxels was also acquired for registration.

### 2.6 MRI processing

#### 2.6.1 Task fMRI analysis

We performed a univariate GLM analysis on the task data. Functional task data were processed using a pipeline developed using software from FSL (Jenkinson et al., 2012), FreeSurfer (Fischl and Dale, 2000), and AFNI (Cox, 1996). Data were corrected for motion with FSL MCFLIRT, high-pass filtered with FSL fslmaths using a sigma of 16.5 volumes, despiked with AFNI 3dDespike, slice time corrected with FSL slicetimer, and spatially smoothed with FSL SUSAN using a 3D Gaussian filter size of 4 mm and median filtering switched off. The time series motion parameters, and the mean signal for eroded (1 mm in 3D) masks of the lateral ventricles and white matter (derived from running FreeSurfer on the T1-weighted image), were added to a GLM as nuisance regressors using FSL's fMRI expert analysis tool (FEAT) version 6.0. Time series statistical analysis was carried out using FILM with local autocorrelation correction. Onsets of each cue (No Cue, Center Cue, and Spatial Cue) and target (Congruent, Incongruent) condition were entered as explanatory variables and convolved with a double-gamma hemodynamic response function. Error trials were modeled separately but not analyzed. The first derivative of the time series motion parameters as well as the original motion parameters, and mean CSF and white matter signals were regressed out as nuisance covariates. The same high-pass filter was applied to the model that was applied to the data. Attention contrasts for the alerting (Center Cue–No Cue), orienting (Spatial Cue–Center Cue), and executive (Incongruent–Congruent) effects were generated for each run for each participant. Co-registration to the T1 image was performed using boundary based registration based on a white matter segmentation of the T1 image (epi_reg in FSL). Contrast images were registered to standard space using FLIRT to apply parameters determined by boundary-based registration of each functional run to the subject's own T1 image and 12-dof linear registration of the subject's T1 to standard Montreal Neurological Institute (MNI) space. Registered contrast images were carried forward into higher-level fixed effect models to generate a single contrast image for each attention contrast for each participant across all runs in both sessions. These contrast images were then fed into a group comparison model, using FMRIB's Local Analysis of Mixed Effects (FLAME) stage 1, to generate a Z statistic image for each contrast.

Cluster thresholding was used to determine if there was significant activation for each attention contrast, while controlling for multiple comparisons. A Z statistic threshold of 2.3 was used to define contiguous clusters. Gaussian random field theory was then used to determine the significance level of each cluster, and a cluster significance threshold of *p* = 0.05 was used to determine significant task clusters.

#### 2.6.2 Resting state fMRI analysis

Resting state functional images were corrected for motion with FSL MCFLIRT, despiked with AFNI 3dDespike, slice time corrected with FSL slicetimer, and spatially smoothed with FSL SUSAN using a 3D Gaussian filter size of 3 mm and median filtering switched off.

rsFC analysis was performed separately for each significant cluster from each attention contrast generated by the group task analysis, as shown in Fig. 1. Each cluster used a separate GLM with FEAT set to use FILM prewhitening for time series statistical analysis. The time series motion parameters, and the mean signal for eroded (1 mm in 3D) masks of the lateral ventricles and white matter (derived from running FreeSurfer on the T1-weighted image), were added to the GLM as nuisance regressors. Due to significant difference in motion between subject groups (with more motion in the HC group — see Supplementary data), and the adverse effect of motion on rsFC analysis, we incorporated volume censoring into the GLM (Power et al., 2014). For each cluster's GLM, the mean resting state fMRI time series across voxels in the cluster was entered as a covariate of interest. This produced a Z statistic rsFC map of how well the cluster's resting state time series modeled every voxel time series throughout the brain.

Co-registration of the rsFC maps to the T1 image was performed using boundary based registration based on a white matter segmentation of the T1 image (epi_reg in FSL). rsFC maps were registered to standard space using FLIRT to apply parameters determined by boundary-based registration of each resting state run to the subject's own T1 image and 12-dof linear registration of the subject's T1 to standard Montreal Neurological Institute (MNI) space. Registered rsFC maps were carried forward into higher-level fixed effect models to generate a single contrast image per cluster for each participant across both sessions. For each cluster, the contrast images were fed into a group comparison model, using FMRIB's Local Analysis of Mixed Effects (FLAME) stage 1, which generated a single Z statistic rsFC image for each task cluster.

Cluster thresholding was used to determine if there were significant rsFC clusters, while controlling for multiple comparisons. A Z statistic threshold of 2.3 was used to define contiguous clusters. Gaussian random field theory was then used to determine the significance level of each cluster, and a cluster significance threshold was used to determine significant rsFC clusters. The overall probability of making a Type I error for the rsFC analysis was set to *p* = 0.05 by adjusting the cluster significance threshold to 0.05 divided by the total number of task clusters used to generate rsFC maps.

We performed a validity check for each task and rsFC cluster that required the maximal value to be located in a voxel with > 50% apriori probability of being grey matter, as determined by FSL tissue priors. Only clusters meeting this criteria are presented in our results.

### 2.7 Resting state network affinity

To relate results of our task and rsFC analysis to attention related resting state networks (RSN), we created rsFC maps for the dorsal attention network (DAN), ventral attention network (VAN), default mode network (DMN), and frontoparietal network (FPN). The DAN is involved in the top down orienting of attention, whereas the VAN is involved in reorienting attention in response to salient sensory stimuli (Fox et al., 2006). The DMN is relevant to attention because of its dynamic relationship with the DAN (Fox et al., 2005), and its involvement in executive deficits in PD (van Eimeren et al., 2009). The FPN is relevant because of its potential role in integrating and/or mediating activity between the DAN and DMN (Spreng et al., 2010, Vincent et al., 2008). The maps were generated using a seed-based approach from coordinates in the literature, summarized in Table S3. MNI coordinates for the DAN, DMN, and FPN were obtained from Power et al. (2011), while Talairach coordinates for the VAN were obtained from Fox et al. (2006) and converted to MNI space. For each network, 3 mm spheres were placed at the network's associated coordinates, and combined to create a single mask. The mean time series within the mask was used to create a network rsFC map in the same way that rsFC maps were created from the task clusters. Clusters from our task and rsFC analysis were then tested to see which of the four RSN they were most closely associated with, by calculating and comparing the weighted mean z-score within the cluster for each RSN, within each subject. Weighted mean z-scores were calculated by summing the voxelwise multiplication of the RSN z-scores within the clusters by the corresponding task (or rsFC) z-scores, then dividing by the sum of task (or rsFC) z-scores. This ensured greater weight was given to the peak voxels within the cluster. The RSN affinity of each cluster was then expressed as a percentage of subjects for which the mean z-score exceeded that of the other three networks.

### 2.8 Correlation analysis

We were interested if there was a linear relationship between activation in task clusters and the rsFC with those regions, and whether this relationship altered in the presence of PD. Such a relationship could occur, for example, if there existed an excitatory or inhibitory influence between regions. As an exploratory analysis we calculated the partial correlation between the attention activation contrasts (alerting, orienting, executive) and rsFC while controlling for age, years of education, and mean RMS head motion during the resting state (see supplementary data). The latter was included to remove effects arising from differences in head motion between groups. Significance of the difference in correlation between groups was tested by transforming the correlation coefficients using Fisher's z-transformation and comparing the z-statistics using the formula by Cohen & Cohen (Cohen and Cohen, 1983). As further exploratory analysis we tested for linear relationships between task activation and behavioral measures. Where significant clusters were identified in the attention activation contrasts, partial correlations were calculated with their respective attention efficiencies for reaction time and accuracy, while controlling for age and years of education. Both exploratory analyses were uncorrected for multiple comparisons, and used a significance threshold of *p* = 0.05.

## 3 Results

### 3.1 Analysis of variance

In keeping with the results from Fan et al. (2005), repeated measures ANOVA of reaction time (RT) showed a significant effect for target condition (executive effect), F(1,42) = 8.83, *p* < 0.01, and cue condition, F(2,41) = 3.82, *p* < 0.05, and there was no significant target × cue interaction, F(2,41) < 0, ns. A significant effect was also observed for alerting, F(1,42) = 7.15, *p* < 0.05, but not orienting, F(1,42) ≤ 0, ns. There was a significant between subject effect for group, F(1,42) = 4.31, *p* < 0.05, with PD having a reaction time of 939 msec (standard deviation [SD] = 204 msec) versus HC having 831 msec (SD = 136 msec). There was no significant between subject effect for age, F(1,42) = 1.89, or years of education, F(1,42) = 4.06, ns. There was also no significant interaction for group × cue, F(2,41) = 2.54, group × target, F(1,42) = 1.49, group × cue × target, F(2,41) < 1, group x alerting, F(1,42) = 3.81, group × orienting, F(1,42) = 2.38, or group × executive, F(1, 42) = 1.49, ns. Table 2 shows the mean group reaction time for each cue-target condition.

We evaluated the possibility that the PD participants were slower to respond solely because of motor impairment by comparing mean response latency for correct Flanker responses on each subject's dominant and nondominant side of motor impairment. By design, half of the Flanker tasks required pushing the left button box and half required pushing the right button box. If elementary motor impairment were a primary reason for slow responses, it should be slower on the predominantly affected side. The mean response latency for all subjects on their side of dominant motor impairment was 935.5 msec (standard deviation [SD] = 193 msec) and for the nondominant side, it was 945.3 msec (SD = 183 msec). A paired *t*-test indicated that these latencies were not significantly different [t(24) = 1.37, *p* = 0.184].

The ANOVA for accuracy showed a significant executive effect, F(1,42) = 7.98, *p* < 0.01, with no significance for the cue effect or cue × target interaction, F(2,41) < 0, ns for both, in keeping with the results from Fan et al. (2005). There was a significant between subject effect for group, F(1,42) = 4.11, *p* < 0.05. There was no significant interaction for group × cue, F(2,41) = 1.86, group × target, F(1,42) = 2.04, group × cue × target, F(2,41) = 2.47, group × alerting, F(1,42) = 2.50, group × orienting, F(1,42) = 3.77, or group × executive, F(1, 42) = 2.04, ns. Table 3 shows the mean group accuracy for each cue-target condition.

### 3.2 PD shows elevated activation in DAN and FPN regions during executive attention task

**Fig. 3:**
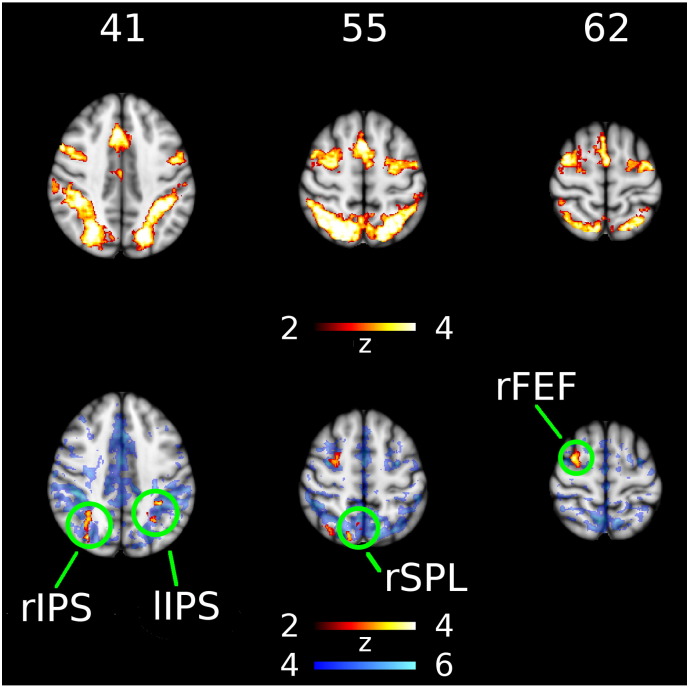
Group differences in executive task activation. Top row shows regions where the response to incongruent stimuli significantly exceeded the response to congruent stimuli for both groups combined. Bottom row shows regions in red where the executive contrast (incongruent–congruent) was significantly increased in Parkinson's disease. Blue background in bottom row shows regions significantly correlated to the dorsal attention network mask. Left side of image is right side of brain. Column label indicates MNI axial coordinate in mm. lIPS, left intraparietal sulcus; rFEF, right frontal eye field; rIPS, right intraparietal sulcus; rSPL, right superior parietal lobule.

Significant group differences were only found for the executive task, which we focus on for the remainder of the report. Across all subjects the executive contrast shows activation in regions of the dorsal attention network (DAN) and frontoparietal network (FPN), including left frontal eye field (lFEF), right frontal eye field (rFEF), left intraparietal sulcus (lIPS), right intraparietal sulcus (rIPS), right superior precuneus, presupplementary motor area, cerebellum, and left and right lateral ventral occipital cortex (Fig. 3 top). These images show where activation in incongruent trials exceeded activation in congruent trials (z-score > 2.3 SD). There were four clusters where the executive contrast for PD exceeded that for HC (Fig. 3 bottom), namely rFEF, lIPS, rIPS, and right superior parietal lobule (rSPL). Coordinates of maximal activation, size, and significance of these clusters are shown in Table 4. Each executive effect cluster had greater RSN affinity with the DAN and FPN, compared with the DMN or VAN (see Table S1 for affinity to each RSN). In particular, the rSPL cluster lay outside the DMN, with greater rsFC to the DAN or FPN in 82.7% of subjects.

### 3.3 Regions of elevated executive activation in PD show reduced connectivity with the DMN

**Fig. 4:**
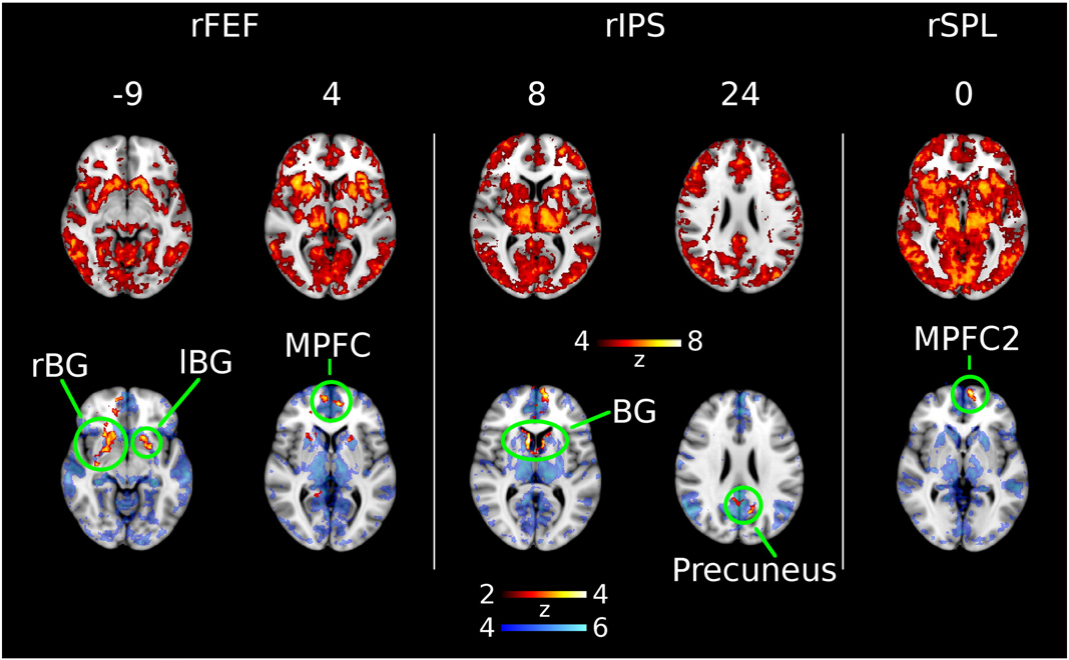
Group differences in resting state functional connectivity (FC) using executive clusters (rFEF, rIPS, rSPL) as seed regions. Top row shows regions significantly correlated with the seed region for combined groups. Bottom row shows regions in red where functional connectivity is significantly reduced in Parkinson's disease. Blue background in bottom row shows regions significantly correlated to the default mode network mask. Left side of image is right side of brain. BG, basal ganglia; lBG, left basal ganglia; lIPS, left intraparietal sulcus; MPFC, medial prefrontal cortex; rBG, right basal ganglia; rFEF, right frontal eye field; rIPS, right intraparietal sulcus; rSPL, right superior parietal lobule.

Each executive effect cluster (rFEF, rIPS, lIPS, rSPL) was used as a seed in a rsFC analysis. The clusters produced similar rsFC maps encompassing a wide network that included the DAN, striatum, thalamus, and mPFC (Fig. 4 top). Two executive effect clusters (rFEF, rSPL) showed reduced rsFC with mPFC in PD (labelled MPFC and MPFC2 in Fig. 4). A significant reduction also occurred between rIPS and mPFC, as seen in Fig. 4, however the a priori grey matter estimate was < 50% at this cluster, and did not meet our criteria for further analysis. rFEF and rIPS also showed reduced rsFC with bilateral striatum (rBG, lBG, BG), and rIPS showed additional reduced rsFC with precuneus in PD. lIPS showed no group differences in rsFC after correcting for multiple comparisons. Coordinates of maximal activation, size, and significance of these clusters are shown in Table 5. Our RSN affinity analysis found three executive effect clusters (located in the DAN/FPN) with reduced rsFC to the DMN. In particular, rIPS showed reduced rsFC to the DMN (precuneus) in 100% of subjects (see Table S2 for affinity of clusters to each RSN). Network maps for the DAN, VAN, DMN, and FPN are shown at the same slices for comparative purposes in the supplementary material (Fig. S2).

### 3.4 Correlation analysis

A significant interaction was found where the relationship between rIPS task activation and rIPS-precuneus resting state connectivity was different between groups. rIPS activation and rIPS-precuneus connectivity were significantly associated in HC (*r*(21) = − 0.55, *p* = 0.02, two-tailed), but had no relationship in PD (*r*(25) = 0.023, *p* = ns). Scatterplots of rIPS activation and rIPS-precuneus connectivity are shown in Fig. S3 of the Supplementary data. The difference between the correlations was significant (*z* = 2.03, *p* = 0.043, two-tailed). There were no other significant correlations between task activation and rsFC for any of the executive effect clusters.

Partial correlations between the executive clusters and executive behavioral measures showed no significant correlations within each individual group. When subjects in HC and PD were pooled, significant correlations were found between the executive reaction time effect and the executive activation contrast at rFEF (*r*(25) = 0.38, *p* = 0.004, two-tailed), and lIPS (*r*(25) = 0.42, *p* = 0.01, two-tailed).

## 4 Discussion

To ignore irrelevant stimuli, both PD and HC engaged a set of regions known to be activated in goal-directed tasks (Cabeza and Nyberg, 2000, Duncan, 2010). PD, however, engaged a subset of this ‘task positive’ network (TPN) to a significantly greater extent. We showed that each region in this subset was more strongly affiliated with the DAN and FPN, compared with the other networks in our affinity analysis, namely the VAN or DMN. Our network affinity analysis also found that three of these regions had reduced rsFC with the DMN in PD. In particular, in PD, rIPS showed significantly reduced rsFC to a region (precuneus) affiliated with the DMN in every subject. Our results, therefore, raise the possibility of an altered relationship between the default mode and TPN, potentially impacting the way in which the executive response is processed in PD.

Our exploratory correlation analysis showed a significant group interaction between rIPS activation and rIPS-precuneus connectivity. While elevated rIPS activation in HC was associated with reduced rIPS-precuneus connectivity, this activation and connectivity was dissociated in PD. This raises the possibility that intrinsic rIPS-precuneus connectivity in PD was reduced to an extent where the precuneus no longer played a role in the regulation of rIPS activation during the executive task. However, as this exploratory analysis was not corrected for multiple comparisons, owing to our small sample size, this result should be treated as preliminary.

Elevated activation in the TPN could arise from more neural resources required to perform the same task, or it could indicate the same resources used for a longer time. The latter, however, is unlikely to explain the increased TPN activation in PD, as the executive reaction time effect was not significantly different between groups. A more likely explanation is that neural processing in the TPN is less efficient in PD, requiring more neural resources. Regions of the TPN, in particular areas in frontal eye field and intraparietal sulcus, are known to exhibit reduced glucose metabolism in PD (Klein et al., 2010, Meles et al., 2015). Klein and colleagues found that both frontal eye field and intraparietal sulcus regions show reduced glucose metabolism in PDD compared to controls and PD without dementia (Klein et al., 2010), though no differences were found between PD without dementia and controls. Eidelberg and colleagues have identified and validated a “PD-related cognitive pattern” in non-demented PD of reduced metabolism in the DAN, precuneus, SMA, pre-SMA, and increased metabolism in the cerebellum, which correlates consistently with performance on executive tasks, and responds to treatment with levodopa (Mattis et al., 2011, Meles et al., 2015). Cortical areas in the PD-related cognitive pattern show a high degree of spatial congruence with our findings of elevated activation in the TPN, supporting the view that neural processing is compromised in these regions.

An important caveat of our results is their potentially confounding by dopaminergic medication, which can impact cognition (Cools, 2006), task activation (Poston et al., 2016), and rsFC in PD. In particular, dopaminergic medication is known to alter striatal rsFC (Prodoehl et al., 2014, Szewczyk-Krolikowski et al., 2014), and could have influenced our result of reduced rsFC to the striatum in PD. Our findings are in agreement with Szewczyk-Krolikowski and colleagues, who found reduced cortico-striatal rsFC in PD off dopaminergic medication compared with HC, including striatal connectivity with dorsolateral prefrontal cortex, medial prefrontal cortex, and precuneus (Szewczyk-Krolikowski et al., 2014). Retesting of subjects on dopaminergic medication resulted in some of these regions increasing rsFC with the striatum, but no regions were observed to decrease striatal rsFC. In contrast to these and our results, Kwak et al. (2010) found an overall increase in cortico-striatal rsFC compared with HC, which upon retesting with dopaminergic medication resulted in an overall decrease in rsFC (Kwak et al., 2010). Many factors can contribute to these inconsistencies among studies (Prodoehl et al., 2014), including differences in disease severity(Luo et al., 2014), seed location in the striatum (Hacker et al., 2012, Kwak et al., 2010), and whether subjects are drug naive or already receiving dopaminergic medication (Esposito et al., 2013). The effect of dopaminergic medication on rsFC between the TPN and DMN is less clear, due to the paucity of reports. In any case, our principal findings are not based simply on comparative levels of rsFC, but rather arise from a changing relationship between rsFC and TPN activation. Higher DMN/TPN rsFC at rest is related to greater rIPS activation in an executive task in controls. However, this relationship does not hold in PD, a disease where there is widespread disruption of resting state networks (Madhyastha et al., 2015c). Changing rsFC dynamics in PD are related to differential task activation. Further tests on and off dopaminergic medication are needed to clarify if these changes are driven by disease processes or medication in PD.

We have shown changes in the interaction between system level networks in PD. Presently, PD has no mechanism-based treatments for attention dysfunction (Svenningsson et al., 2012). Further elucidation of system level attention network changes, and their underlying genetic and neuromodulatory mechanisms, will aid development of treatments to improve quality of life in PD. The results here motivate looking at rsFC as a marker of physiological wellness and system specific alterations.

## Funding sources

NIH RC4 NS073008, T32 AG0000258, P50 NS062684.

## Figures and Tables

**Table 1:** Sample demographics.

	PD	HC
N	25	21
Age (years)	66.0 (10.1)	61.9 (10.0)
Gender (number of males)	18	9
Hoehn & Yahr	2.0 (1–2.5)	
UPDRS Part I	10.0 (5.7)	
UPDRS Part II	8.8 (5.3)	
UPDRS Part III *	23.6 (8.7)	0.81 (1.4)
UPDRS Part IV	2.0 (3.7)	
Time since symptom onset (years)	8.5 (4.8)	
MoCA	26.4 (2.1)	27.3 (2.0)
Education (years)	16.2 (2.1)	15.9 (2.4)
Dopaminergic antiparkinsonian medication (number of subjects)	23	
Handedness (right)	21	19
Dominant side of motor symptoms	18 right, 7 left	

Means (SD). *UPDRS Part III (motor subscale) done ON dopaminergic medication. Groups differ significantly on the UPDRS Part III, t(44) = 11.82, *p* < 0.001, and in proportion male t(44) = 2.05, *p* = 0.047. They do not differ significantly in education, age, or MoCA score. HC, Healthy controls; MoCA, Montreal Cognitive Assessment score; PD, Parkinson disease; UPDRS, Unified Parkinson's Disease Rating Scale.

**Table 2:** Mean reaction time (and SD) in milliseconds.

	No cue	Center cue	Spatial cue	Mean
Congruent				
PD	**926 (182)**	874 (163)	**816 (166)**	872 (174)
HC	**821 (121)**	782 (115)	**717 (116)**	773 (123)

Incongruent				
PD	**1060 (209)**	1016 (198)	**942 (218)**	1006 (211)
HC	**937 (143)**	911 (140)	**820 (131)**	889 (145)

Mean				
PD	993 (205)	945 (193)	879 (202)	939 (204)
HC	879 (144)	847 (142)	769 (133)	831 (146)

Significant group differences, according to post hoc Scheffé tests (*p* < 0.05), are shown in bold. HC, healthy controls; PD, Parkinson's disease.

**Table 3:** Mean accuracy (and SD) as a percentage of trials.

	No cue	Center cue	Spatial cue	Mean
Congruent				
PD	**97 (3.8)**	98 (2.6)	98 (2.9)	98 (3.1)
HC	**99 (1.6)**	99 (1.7)	99 (1.7)	99 (1.7)

Incongruent				
PD	97 (3.5)	**95 (4.3)**	97 (3.4)	96 (3.8)
HC	98 (2.0)	**98 (2.7)**	98 (2.8)	99 (2.5)

Mean				
PD	97 (3.6)	97 (3.5)	98 (3.1)	97 (3.5)
HC	99 (1.8)	98 (2.2)	98 (2.2)	98 (2.2)

Significant group differences, according to post hoc Scheffé tests (*p* < 0.05), are shown in bold. HC, healthy controls; PD, Parkinson's disease.

**Table 4:** Characteristics of significant clusters for executive contrast.

Cluster name	Voxels	p	*Z*-MAX	MNI coordinates (mm)	Anatomical regions
rIPS	2163	1.19E-06	3.82	32, − 72, 40	Right lateral occipital cortex
lIPS	1149	0.0011	3.53	-33, − 42, 41	Left superior parietal lobule, left angular gyrus
rFEF	1117	0.00141	3.52	30, − 2, 62	Right superior frontal gyrus
rSPL	735	0.031	3.28	14, − 71, 55	Right precuneus cortex, right superior parietal lobule

lIPS, left intraparietal sulcus; rFEF, right frontal eye field; rIPS, right intraparietal sulcus; rSPL, right superior parietal lobule.

**Table 5:** Characteristics of significant clusters for functional connectivity analysis.

Seed	Cluster name	Voxels	p	*Z*-MAX	MNI coordinates (mm)	Anatomical regions
rFEF	rBG	3416	1.28E-09	3.91	17, 19, − 6	Right caudate
	lBG	1384	0.00023	3.44	− 17, 16, − 9	Left putamen
	MPFC	859	0.0124	3.55	− 8, 53, 4	Paracingulate gyrus
rIPS	BG	4603	4.63E-11	4.18	9, 13, 8	Caudate
	Precuneus	1403	0.000599	3.37	− 10, − 64, 25	Precuneus
rSPL	MPFC2	1078	0.00955	3.86	− 8, 64, 0	Frontal pole

BG, basal ganglia; lBG, left basal ganglia; lIPS, left intraparietal sulcus; MPFC, medial prefrontal cortex; rBG, right basal ganglia; rFEF, right frontal eye field; rIPS, right intraparietal sulcus; rSPL, right superior parietal lobule.

## Appendix A Supplementary data

Supplementary material Image 1
