# Supplementary material for: Executive attention networks show altered relationship with default mode network in PD
Source: Neuroimage Clin. 2016 Nov 5;13:1–8. doi: 10.1016/j.nicl.2016.11.004 (PMC5121155; doi:10.1016/j.nicl.2016.11.004)
Supplement: Supplementary file 1 — Supplementary material [file mmc1.docx]

Supplementary Data

## Head Motion Assessment

Each task and resting state fMRI run was motion corrected with FSL MCFLIRT, using the “-rmsrel” option to output the mean RMS deviation between transformation matrices in millimeters. The deviations were averaged over six task and two resting state runs for each subject, and converted to a z-score based on the mean and standard deviation across all subjects. Histograms of the mean RMS head motion were plotted for task and resting state (Figure S1).


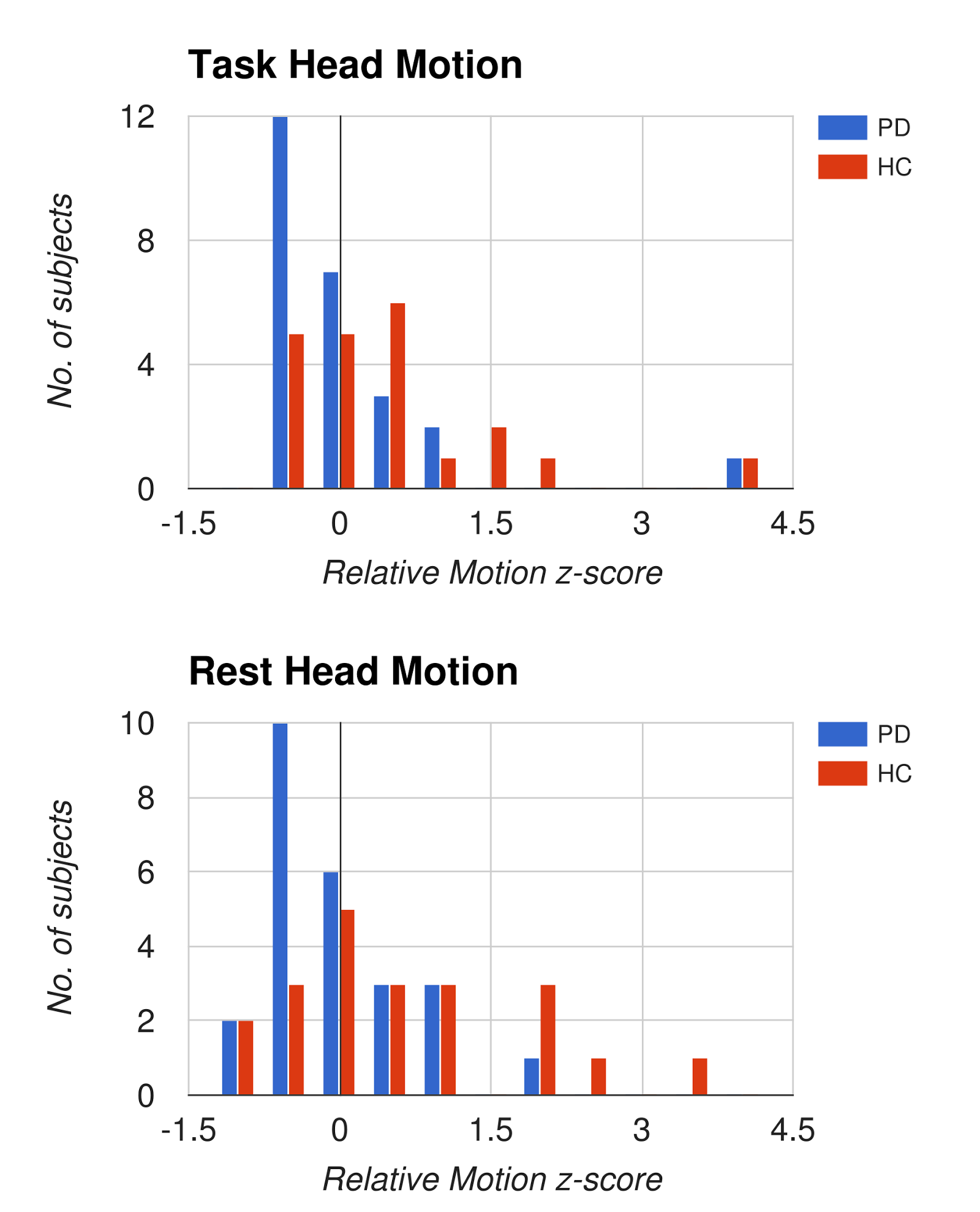


Figure S1. Histogram of mean RMS task (top) and resting state (bottom) head motion. HC, healthy controls; PD, Parkinson’s disease.

In the task, there was no significant difference in head motion between groups (PD: 0.1±0.07 mm; HC: 0.14±0.08 mm; *p*=0.13 two-tailed t-test). We reanalyzed the task data without the two subjects showing maximal movement (1 PD, z=3.51; 1 HC, z=3.53), and the results were essentially the same. The results reported include these two subjects.

In the resting state, there was a significant difference in head motion between groups, with HC showing *more* movement than PD (PD: 0.1±0.04 mm; HC: 0.14±0.07 mm; *p*=0.02 two-tailed t-test). To limit the effect of head motion on our results we used volume censoring in our GLM analysis, and included RMS relative head motion z-scores as a confound in our correlation analyses.

**Resting State Networks**


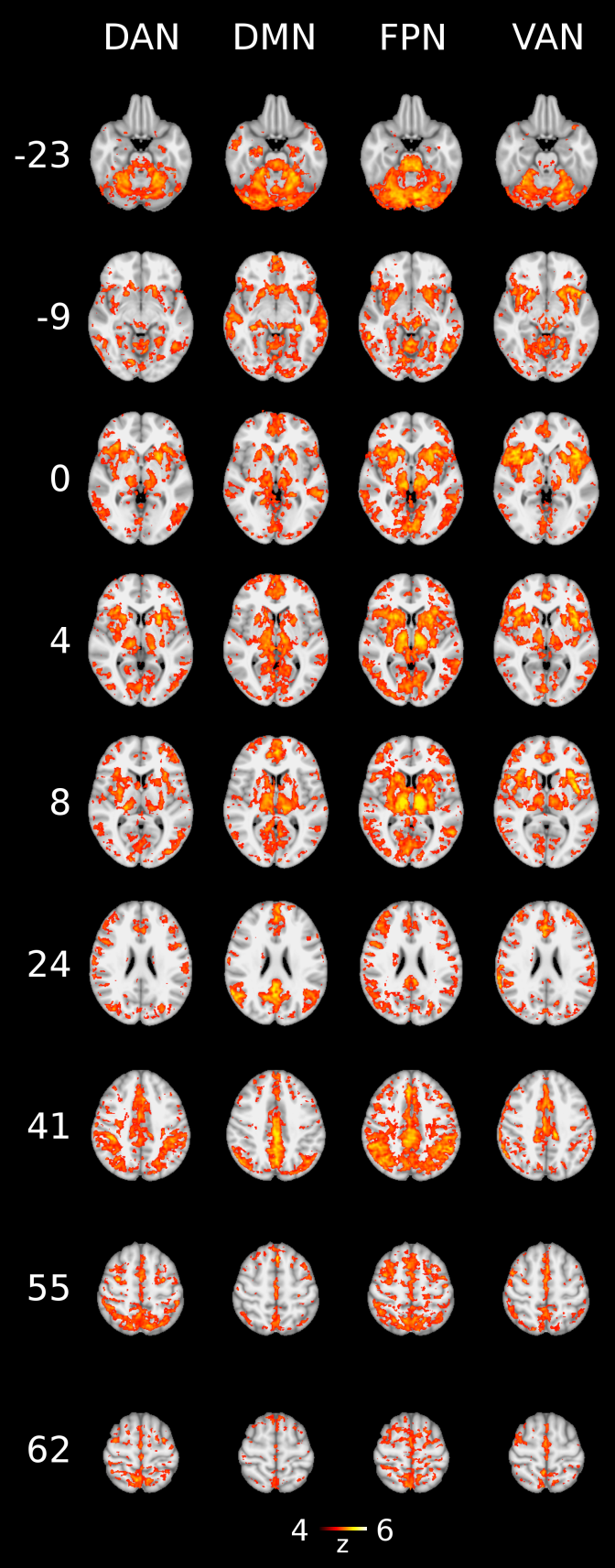


Figure S2. Resting state networks at axial slices used in the main report. Left column indicates MNI axial slice in mm. DAN, dorsal attention network; DMN, default mode network; FPN, frontoparietal task control network; VAN, ventral attention network


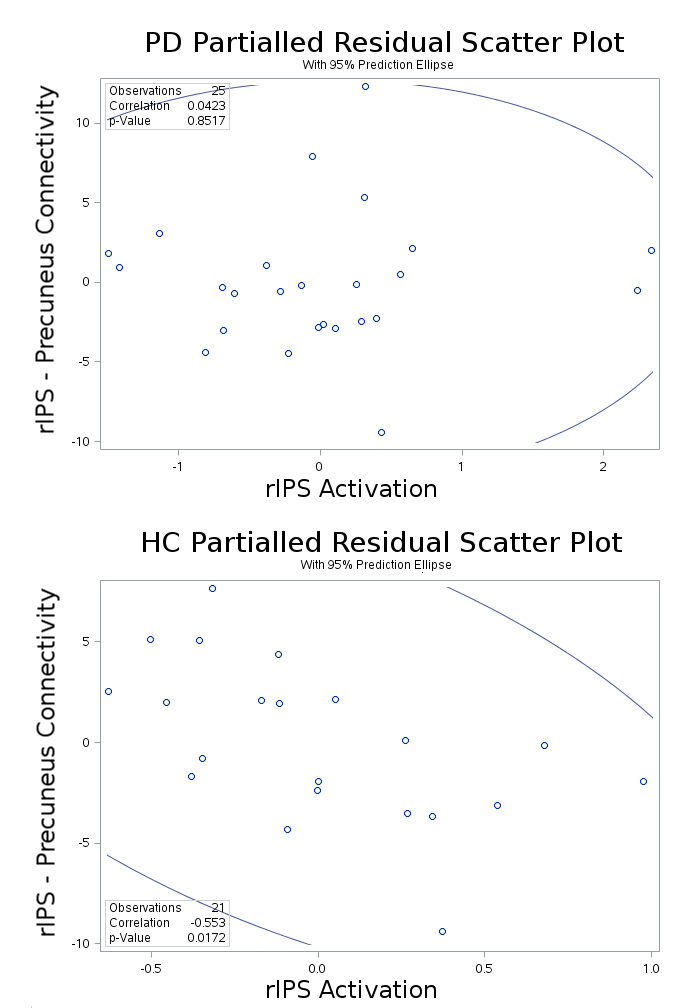


Figure S3. Partialled residual scatter plots of rIPS-Precuneus connectivity versus rIPS activation in PD (top) and HC (bottom). rIPS, right intraparietal sulcus. HC, healthy controls; PD, Parkinson’s disease.

###### Table S1. Percentage of subjects with greatest connectivity to the four resting state networks

| **Cluster name** | **DAN** | **DMN** | **FPN** | **VAN** |
| --- | --- | --- | --- | --- |
| rIPS | 41.3 | 2.2 | 52.2 | 4.3 |
| lIPS | 47.8 | 0.0 | 52.2 | 0.0 |
| rFEF | 45.7 | 2.2 | 47.8 | 4.3 |
| rSPL | 37.0 | 15.2 | 45.7 | 2.2 |

Clusters: lIPS, left intraparietal sulcus; rFEF, right frontal eye field; rIPS, right intraparietal sulcus; rSPL, right superior parietal lobule. Networks: DAN, dorsal attention network; DMN, default mode network; FPN, frontoparietal task control network; VAN, ventral attention network.

###### Table S2. Percentage of subjects with greatest connectivity to the four resting state networks

| **Seed** | **Cluster name** | **DAN** | **DMN** | **FPN** | **VAN** |
| --- | --- | --- | --- | --- | --- |
| rFEF | **rBG** | 10.9 | 30.4 | 39.1 | 19.6 |
|  | **lBG** | 15.2 | 17.4 | 34.8 | 32.6 |
|  | **MPFC** | 8.7 | 65.2 | 8.7 | 17.4 |
| rIPS | **BG** | 8.7 | 43.5 | 34.8 | 13.0 |
|  | **Precuneus** | 0.0 | 100.0 | 0.0 | 0.0 |
| rSPL | **MPFC2** | 10.9 | 76.1 | 8.7 | 4.3 |

Clusters: BG, basal ganglia; lBG, left basal ganglia; lIPS, left intraparietal sulcus; MPFC, medial prefrontal cortex; rBG, right basal ganglia; rFEF, right frontal eye field; rIPS, right intraparietal sulcus; rSPL, right superior parietal lobule. Networks: DAN, dorsal attention network; DMN, default mode network; FPN, frontoparietal task control network; VAN, ventral attention network.

###### Table S3. MNI coordinates used for the DAN, DMN, FPN, and VAN resting state network affinity analysis

| **Resting State Network** | **MNI coordinates (mm)** |
| --- | --- |
| DAN | -26 -65 52  -29 -5 55  28 -65 51  31 -5 54  43 -36 46  -45 -37 48 |
| DMN | 1 -51 29  -1 61 22  -48 -66 34  53 -61 35  -65 -23 -9  61 -21 -12 |
| FPN | 46 28 31  -44 27 33  44 8 34  -42 7 36  54 -44 43  -53 -50 39  32 -59 41  -32 -58 46 |
| VAN | 31 14 -3  62 -41 37  -43 13 -4  32 50 24  45 45 -3  52 19 29  6 27 48  57 -33 -10  -63 -49 22  4 -50 57  10 10 64  45 -13 -11  8 -25 46 |

DAN, dorsal attention network; DMN, default mode network; FPN, frontoparietal task control network; VAN, ventral attention network
